# 3D Non-Rigid Alignment of Low-Dose Scans Allows to Correct for Saturation in Lower Extremity Cone-Beam CT

**DOI:** 10.1109/access.2021.3079368

**Published:** 2021-05-11

**Authors:** JENNIFER MAIER, ANDREAS MAIER, BJOERN ESKOFIER, REBECCA FAHRIG, JANG-HWAN CHOI

**Affiliations:** 1 Pattern Recognition Laboratory, Department of Computer Science, Friedrich-Alexander-Universität Erlangen-Nürnberg, 91058 Erlangen, Germany; 2 Machine Learning and Data Analytics Laboratory, Department of Computer Science, Friedrich-Alexander-Universität Erlangen-Nürnberg, 91052 Erlangen, Germany; 3 Siemens Healthcare GmbH, 91301 Forchheim, Germany; 4 Division of Mechanical and Biomedical Engineering, Graduate Program in System Health Science and Engineering, Ewha Womans University, Seoul 03760, South Korea

**Keywords:** Automatic exposure control, C-arm cone-beam CT, detector saturation, lower extremity, overexposure artifacts, non-rigid reconstruction, X-ray imaging and computed tomography

## Abstract

Detector saturation in cone-beam computed tomography occurs when an object of highly varying shape and material composition is imaged using an automatic exposure control (AEC) system. When imaging a subject’s knees, high beam energy ensures the visibility of internal structures but leads to overexposure in less dense border regions. In this work, we propose to use an additional low-dose scan to correct the saturation artifacts of AEC scans. Overexposed pixels are identified in the projection images of the AEC scan using histogram-based thresholding. The saturation-free pixels from the AEC scan are combined with the skin border pixels of the low-dose scan prior to volumetric reconstruction. To compensate for patient motion between the two scans, a 3D non-rigid alignment of the projection images in a backward-forward-projection process based on fiducial marker positions is proposed. On numerical simulations, the projection combination improved the structural similarity index measure from 0.883 to 0.999. Further evaluations were performed on two *in vivo* subject knee acquisitions, one without and one with motion between the AEC and low-dose scans. Saturation-free reference images were acquired using a beam attenuator. The proposed method could qualitatively restore the information of peripheral tissue structures. Applying the 3D non-rigid alignment made it possible to use the projection images with inter-scan subject motion for projection image combination. The increase in radiation exposure due to the additional low-dose scan was found to be negligibly low. The presented methods allow simple but effective correction of saturation artifacts.

## I. INTRODUCTION

The advantages of C-arm cone-beam computed tomography (CBCT) systems with large flat-panel detectors are manifold. Besides offering high spatial resolution and bone contrast, their flexibility enables scanning on almost arbitrary trajectories. This makes it possible to perform an acquisition of a subject’s knees in a standing weight-bearing position in order to analyze knee joint mechanics under real-life loading conditions. However, this configuration also causes some issues, such as patient motion and overexposure artifacts. While the former was already addressed in previous works [1]–[3], the latter is the topic of the presented work.

**FIGURE 1.**
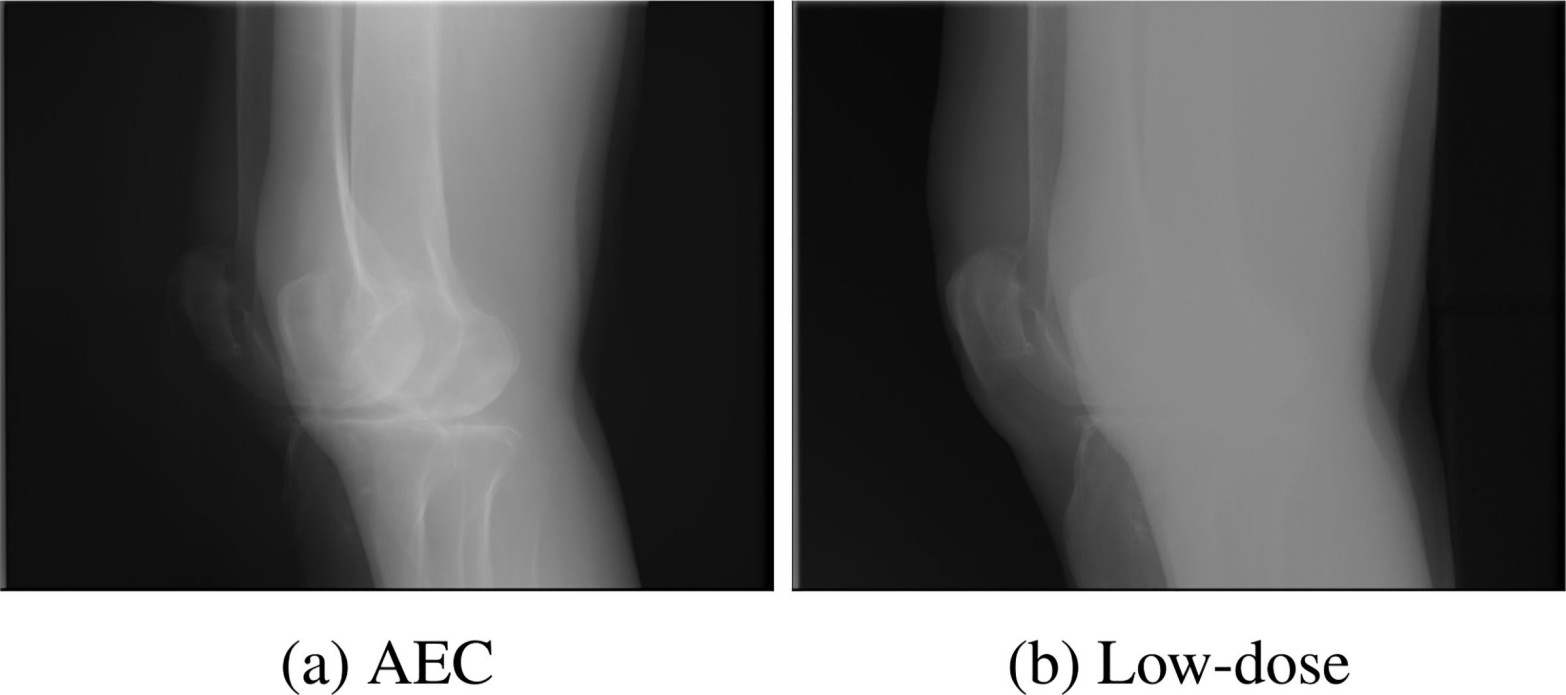
Example projection images of (a) automatic exposure control and (b) low-dose acquisition of both knees. Saturation artifacts in the AEC scan are visible in the anterior and posterior region of the knees. In the low-dose scan, these regions are properly depicted but photon starvation leads to information loss inside the knee joint.

Overexposure artifacts in knee imaging occur because of the irregular shape of the knees. In the lateral view, the X-rays have to pass through both femur bones, so the automatic exposure control (AEC) system increases the tube voltage and tube current in order to ensure the visibility of those structures. Simultaneously, the high output energy leads to the saturation of the detector in regions where the rays are not attenuated or only slightly attenuated. For example, this happens at the skin-air interface at the border of the knees. Figure 1 shows an example projection image in which this effect is apparent. Consequently, it is impossible to accurately reconstruct structures situated in this region, such as parts of the patella, the patellar tendon, and the quadriceps tendon, which are relevant for clinical diagnosis.

Existing approaches try to either prevent or correct detector saturation. For example, hardware solutions for overexposure prevention apply multi-gain read-out technology to extend the dynamic range, but at the cost of reducing the frame rate or resolution [4]. Alternatively, they use analog non-linear transformation-based tone mapping that has, thus far, only been evaluated on simulated data [5]. Furthermore, a bowtie filter attenuating the X-rays pre-patient depending on their distance to the iso-center was proposed; however, this only works well when assuming a cylindrical or ellipsoidal object [6]. Software-based correction of saturation most often makes use of an estimation model to extrapolate the missing data [7]–[11]. Knaup *et al.* [12] investigated how intentional overexposure can be leveraged to achieve better low-contrast visibility in inner object regions. The unwanted saturation at the borders was also corrected using a simple extrapolation. While this works well for simple object shapes, most methods fail when correcting more complex structures consisting of multiple materials. Huang *et al.* [13], [14] propose a recovery method called mixed one-bit compressive sensing, which yields promising results on numerical simulations but needs prior knowledge of the location of saturated pixels. Rausch *et al.* [15] used a depth camera to reconstruct the skin border requiring external hardware and manual input.

We propose to combine the projection images of two CBCT scans in order to correct overexposure artifacts, where one of the scans is acquired using the AEC protocol and the second using a low but constant dose. While the AEC scan contains saturated regions owing to overexposure, these regions are correctly represented in the low-dose scan as visualized in the projection image in Fig. 1b. The low-dose scan instead suffers from photon starvation in the inner leg regions, but this can be ignored because these regions are not used for reconstruction. By replacing the content of the saturated regions of the AEC scans with that of the low-dose scan, saturation corrected projection images are obtained.

The idea of using an additional low-dose scan was initially proposed in a previous study [16]. However, the presented method suffered from some issues that are addressed in this work. First, the method for combining the AEC and low-dose projection images is revised. In the previous study, intersections between the intensities of the AEC and low-dose scans were searched row-wise and were used to determine the overexposed areas. However, it is unlikely that the scaling of the two scans is similar enough to allow the application of this method. We therefore present a method based on the histogram of the AEC scan in order to find a threshold separating background pixels and overexposed pixels from the properly imaged pixels. Second, an evaluation of our method on a numerical phantom is presented. This has the advantage that a reference scan using a higher range detector that does not suffer from overexposure can be simulated in order to allow for a quantitative analysis of the results. Third, we address the concern that motion between the scans leads to a mismatch of the projection images and thereby hinders a successful projection image combination. We propose to apply non-rigid alignment to the low-dose scan in a backward-forward-projection process in order to obtain projection images that match those of the AEC scan well and can therefore be used for projection image combination. Finally, a dose estimation is performed to determine the expected radiation increase induced by the additional low-dose scan.

## II. METHODS

The inputs to our processing pipeline are the line integral projection images and the geometries of one AEC CBCT scan and one low-dose CBCT scan of the knees. The combination of the projection images for exposure correction comprises the following steps: first, the overexposed regions in each AEC projection image are determined and used to compute binary masks for the AEC and low-dose scans. In case of subject motion between the AEC and low-dose scans, a registration process between the AEC and low-dose scans based on small metallic markers on the knees is applied. Before combining the masked AEC and low-dose projection images, the low-dose images are scaled to achieve a smooth transition at the mask margins. The final combined projection images are used to reconstruct the saturation-corrected volume. The following sections describe the processing steps in more detail.

### A. MASK GENERATION

The pixels in the saturated regions of the AEC projection images have approximately the same values as those pixels where the X-rays only passed through air before reaching the detector, which will be called “background” pixels in the following descriptions. The saturated regions of each projection image are determined based on a background threshold that is obtained from the histogram of the projection image. All values below this threshold are defined as background or overexposed pixels, while all values above are defined as non-saturated pixels.

**FIGURE 2.**
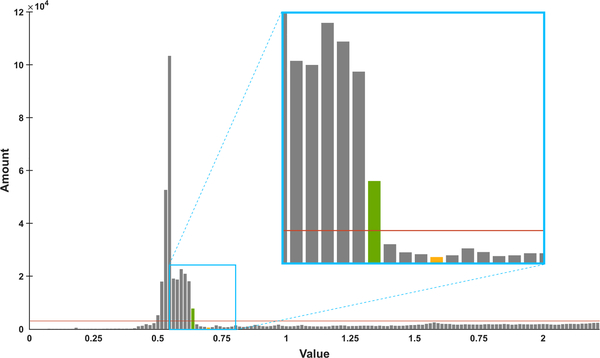
Visualization of the automatic histogram-based threshold detection for mask generation for an example projection image. In the lower quarter of the histogram, the rightmost bin with a higher amount than the average bin amount (orange line) is detected (green bar). The value of the first minimum to the right of this bin (yellow bar) is defined as the threshold that divides the foreground pixels from the background and saturated pixels.

The process for finding the threshold is described with the aid of Fig. 2, which shows the histogram of an example projection image. For each line integral projection image of the AEC scan, the histogram is computed with a fixed bin width of 0.02. Consequently, the number of bins depends on the pixel value range of the projection image. In the histogram, the background and overexposed pixels are represented as a bell-shaped peak in the lower value region. For this reason, only the lower quarter of the histogram is considered. To find the bell-shaped background peak, the mean amount of the bins of the lower quarter of the histogram is computed (orange line in Fig. 2) and all histogram bins with amounts above this mean amount are defined to belong to the background peak. Starting at the rightmost bin of these background peak bins (green bar in Fig. 2), the next minimum of the histogram with a higher bin value is determined (yellow bar in Fig. 2). The bin value at this minimum is defined as threshold for separating saturated and non-saturated pixels in this projection image. To create the AEC mask for each projection image, all values above the threshold are set to one and all values below are set to zero. If there are small one-valued regions with a width of less than five pixels in a row of the mask image, they are set to zero in order to achieve a less speckled mask. The low-dose mask is then obtained by inverting the AEC mask.

In some cases, there is more than one such bell-shaped peak present in the lower quarter of the histogram. This can happen if the CT table the patient is lying on is visible in the background or if there is only a small background region and most of the projection image is filled with the legs of the subject such that the background peak is not distinctively higher than the foreground information. In the former case, this second peak should be counted as belonging to the background, whereas in the latter, it should not be included in the background region. To differentiate between the two cases, a limit of 10 bins between the two peaks is defined. If the distance of the two peaks is below this limit, they are defined to both belong to the background. If the distance is above the limit, the second peak is defined not to belong to the background.

### B. 3D NON-RIGID ALIGNMENT

**FIGURE 3.**
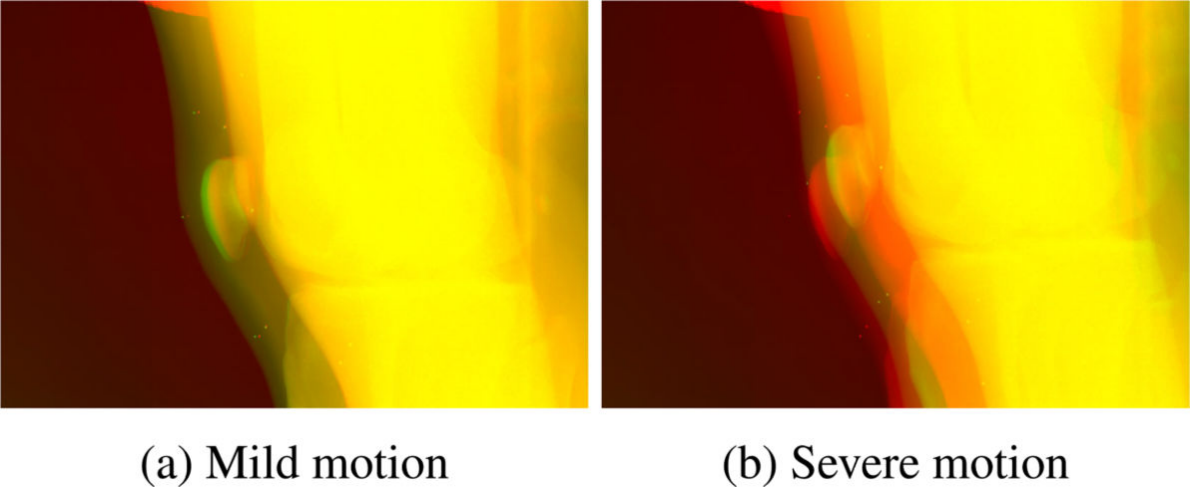
Overlay of example projection images of the AEC scan and the low-dose scan with (a) mild and (b) severe motion between AEC and low-dose scans. The AEC projection image is depicted in red, the low-dose projection images in green, and similar pixels are shown in yellow.

If the patient moves between the AEC and low-dose scans, the masks generated from the AEC scan do not match with the low-dose projection images anymore. An example overlay of projection images where this is visible is shown in Fig. 3. The AEC projection image is depicted in red, the low-dose projection image in green, and all similar pixels are shown in yellow. Two cases where a mild (Fig. 3a) and a severe (Fig. 3b) motion occurred between the two scans are presented. In such cases, an additional registration process needs to be applied in order to be able to properly combine the projection images.

Although the motion appears to only cause a shift between the projections shown in Fig. 3, a closer look at the data reveals that the projections cannot simply be registered by an affine transformation. The reason for this is that the position of the legs can change non-rigidly between exposures, e.g., if the opening angle of the legs changes or if one of the legs is rotated slightly further in or out. Consequently, the X-rays of the low-dose scan are attenuated by different materials on their path leading to different content in the projection images. The motion therefore needs to be corrected in the 3D domain. For this purpose, we apply a non-rigid deformed reconstruction to the low-dose projections to obtain a reconstructed volume that matches the conventionally reconstructed AEC volume. Then, the deformed low-dose reconstruction is forward-projected using the system geometry in order to obtain projection images that are suitable for the combination with the AEC projection images. The details of this two-step process are explained in the following paragraphs.

The deformed 3D reconstruction is based on small metallic markers that are attached to the patient’s legs. Eleven 1-mm-diameter tantalum markers are placed near the knee joint such that their projections are visible in the projection images without overlapping. As metal has a high absorption coefficient, the markers can easily be recognized in the projection images. We apply an automatic tracking of the projected marker positions that was proposed in a previous work [17]: using fast-radial-symmetry-transform (FRST) [18], the circular projections of the markers are identified in all projection images. The FRST-transformed projection images are then back-projected using the system geometry to generate a blurry 3D blob for each marker. These blobs are binarized and their centroids are automatically obtained using connected-component labeling in 3D. The resulting 3D positions of the 11 markers in the AEC and low-dose scans are then used as the input for 3D deformed reconstruction. To prevent metal artifacts in the final reconstructed volumes, the projections of the markers are removed from all projection images before further processing using a spectral interpolation method proposed by Berger *et al.* [17].

To obtain a non-rigidly deformed volume, moving least squares (MLS) deformation is applied during the back-projection [19], [20]. This approach was proposed and is described in more detail in a previous work where subject motion during standing CBCT scans of the knee was corrected [21].

In MLS deformation, a scene is controlled using a set of *n* control points whose initial positions **p** and target positions **q** are known. Using these control points, for each point **v** in the volume, a linear transformation *f* (**v**) is computed that minimizes the distance between the target positions and the transformed initial positions weighted depending on the considered point **v**. In the presented case, the *n* = 11 control points are the 3D positions of the metal markers, where the low-dose positions are the initial positions **p** and the AEC positions are the target positions **q**.

The MLS deformation is then incorporated in the CT volume reconstruction. During conventional back-projection, the value at a certain 3D position of the output volume is computed by forward-projecting the point onto the detector for each projection using the system geometry, reading out the respective projection image at the projected position, and adding the readout value to the value at the 3D position. To obtain the deformed reconstruction, the 3D position is transformed by MLS deformation before forward-projecting it onto the detector but the readout value is still added at the original position.

The resultant deformed low-dose reconstruction is then virtually forward-projected and scaled using the original projection images in order to obtain the registered projection images. In the software framework CONRAD [22], virtual projection images are generated for each acquisition angle using the system geometry of the original scan. These aligned projection images are then used for the combination with the AEC scan.

### C. PROJECTION COMBINATION AND RECONSTRUCTION

The AEC projection images and the low-dose projection images are masked by multiplying them with the masks generated from the AEC scan, as explained in Section II-A. The low-dose projections to be masked are the original projection images in cases without motion between the scans and the projection images generated by forward projection of the deformed reconstruction if the patient moved between the scans (see Section II-B).

The masked AEC and low-dose projection images are combined by adding them together. However, given their different acquisition voltages, the projection images of AEC and low-dose scans also have different value ranges, which will lead to a visible jump in intensity values at the mask border. Low-energy rays are more likely to be absorbed by the pervaded material than high-energy rays [23]. Consequently, comparably fewer photons reach the detector in the low-dose scan than in the AEC scan, even though similar tissue is imaged. In order to guarantee a smooth transition before adding the masked projection images, the value jump at the mask border is corrected by scaling the masked low-dose scan row-wise to have the same value as the AEC scan at the mask border. This row-wise scaling might introduce minor intensity jumps between rows. However, no noticeable artifacts are expected from this in the resultant reconstructions as these differences occur along the rotation axis of the scan.

Note that the masking and combination process only has to be performed for projection images where overexposure actually occurred. The C-arm trajectory covers 180° plus the fan opening angle. In the recorded setting, the rotation starts at a lateral view on the knees, rotates to the anterior-posterior view, and finishes at the opposite lateral view. The AEC system only elevates the tube outputs along the lateral direction because more radiation is needed to penetrate through two thick femurs, which leads to overexposure at the border of the knees in these projections. In the anterior-posterior direction where the legs do not overlap, the tube output is reduced and consequently no overexposure occurs. Therefore, the proposed combination for overexposure correction is only applied to the first and last 80 projection images covering the lateral view, while the other projection images covering the anterior-posterior view are left unaltered.

The final projection images are then reconstructed to 3D volumes using filtered back-projection. The filtering pipeline consists of a cosine weighting, Parker weighting, truncation correction, Shepp-Logan ramp filtering, and conventional back-projection.

### D. RADIATION DOSE ESTIMATION

**FIGURE 4.**
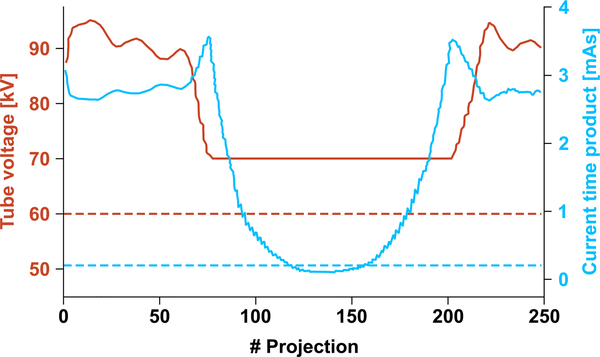
Tube voltage (orange) and current-time product (blue) of the C-arm CT system during real scans. Solid lines represent the automatic exposure-controlled tube outputs, while dashed lines are the constant tube outputs of the low-dose scan.

The effective radiation dose of the low-dose and over-dose scans was estimated using the commercial Monte Carlo simulation package PCXMC2.0 (Radiation and Nuclear Safety Authority, Helsinki, Finland) [24]. To match the X-ray spectrum of the simulation to that of the actual C-arm CT system, source filters of PCXMC that equalize the half-value layer (HVL) values of the two X-ray spectra were set up. The HVL value of the C-arm CT system was 2.9 mm Al measured at a tube voltage of 70 kVp [25]. The values of tube outputs (kilo voltage and current-exposure time product) were imported as a function of the gantry angle from the scan of *in vivo* human subject knee data (see Fig. 4). To perform a patient-specific dose calculation, the subjects’ dimensions were imported as well. The effective dose was calculated based on the tissue-weighting coefficients recommended by the International Commission on Radiological Protection (ICRP) [26].

## III. EXPERIMENTAL EVALUATION

The proposed pipeline for combining AEC and low-dose projection images is evaluated in three parts. First, CT scans of a numerical phantom are simulated to evaluate the feasibility of the proposed approach in an optimal setting. Second, actual AEC and low-dose scans of one patient without motion between the two scans are acquired in order to evaluate solely the projection image combination. Third, to evaluate the proposed registration process, a second subject is scanned using the AEC protocol; then, the subject intentionally moved before the low-dose scan is acquired. The specifics of the three evaluation parts are presented in the following subsections.

### A. NUMERICAL SIMULATION

The proposed algorithm is evaluated on numerical model-based simulations. Real data acquisitions are limited by the dynamic range of the detector. By contrast, during simulations, a detector with higher dynamic range can be modeled and used to generate a reliable ground truth. Using this, a quantitative evaluation of the proposed software-based method can be performed. For the simulation, the XCAT-model developed by Segars *et al.* [27] was used. The modeled knee joint consists of the femur, tibia, patella, and fibula bones with bone marrow inside and fat tissue around. To simulate the absorption behavior of the various materials, their volumes were rendered individually and then forward-projected on the detector using polychromatic X-ray spectra.

The polychromatic X-ray spectra were simulated depending on the peak kilovoltage (kVp) and the current-time product (in mAs). The spectrum for the low-dose scan was generated with a peak kilovoltage of 60 kVp and a current-time product of 0.21 mAs. To ensure the visibility of dense structures, the high-dose scan had a peak kilovoltage of 120 kVp and a current-time product of 2.5 mAs. Hence, the spectra had a constant initial intensity *I*_0*,low*_ ≪ *I*_0*,high*_.

The simulated polychromatic detector had a size of 640 × 480 pixels with a spacing of 0.616 mm and a bit depth of 14 bits. The simulated C-Arm rotated on a circular trajectory with a source detector distance of 1198 mm. On this trajectory, 248 projections were simulated with an angular increment of 0.8 degrees after each projection. For the simulation of detector pixel saturation, the maximum measurable intensity *I*_*max*_ was defined based on the two initial intensities *I*_0*,low*_ and *I*_0*,high*_:

(1) $$I_{max}=(1-t)\cdot I_{0,low}+t\cdot I_{0,high},$$

where *t* = 0.2 is empirically chosen. As the dynamic range of the simulated detector was 14 bits, each bin for quantization had a width of *I*_*max*_*/*2^14^.

For each ray cast to the detector, the absorption by the materials on its path was computed resulting in an attenuated intensity *I* measured by the detector. To simulate overexposure and saturation, all resulting intensities above *I*_*max*_ were set to *I*_*max*_ before quantization. The binned intensities were divided by the respective *I*_0_ before computing the line integral, −ln *I/I*_0_.

The above described process fulfills two purposes. First, border regions of the knee at the air-skin interface are overexposure in the high-dose scan. If the initial intensity *I*_0*,high*_ of the high-dose spectrum is only slightly attenuated by thin skin material, the resulting intensity is still above *I*_*max*_ and is cut off before quantization. Second, in the low-dose scan, photon starvation occurs in the dense regions of the knee. This happens if the low-dose spectrum’s intensity *I*_0*,low*_ is completely attenuated by the material on the ray’s path. Consequently, no signal is measured at the detector resulting in missing information in those regions.

Another projection dataset was simulated to generate a reference reconstruction for comparison of the result of the proposed algorithm. To this end, the same spectrum as for the overexposure case with the same bin width as before was used. However, the overexposure step of cutting off all values above *I*_*max*_ is skipped. In essence, this means that a detector with higher bit depth was simulated, but the bin width was kept the same to make the results comparable.

**FIGURE 5.**
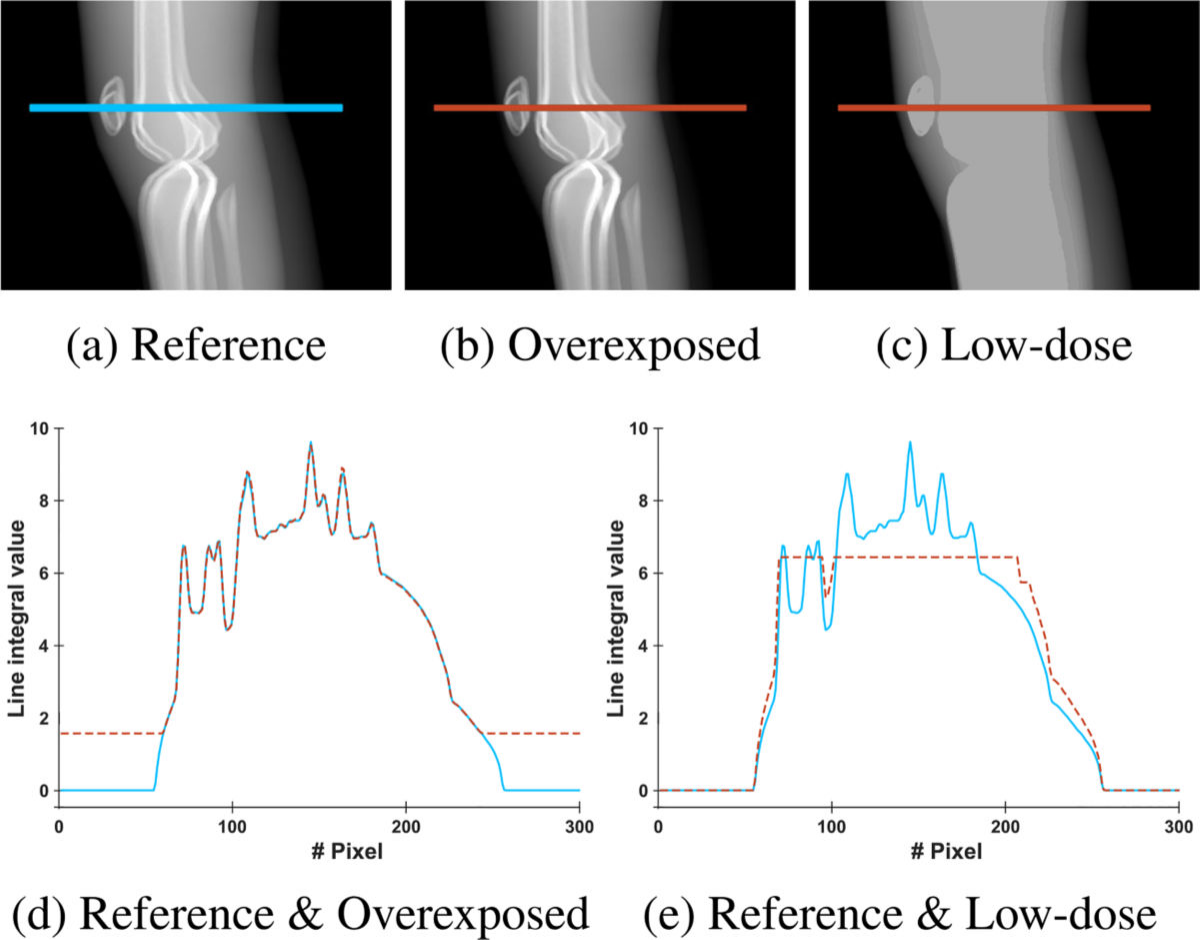
Example projection images of the presented simulation. Compared to (a) the reference image, (b) the overexposed image shows saturation artifacts and (c) the low-dose image shows photon starvation. Line profile plots along the lines drawn in the images are shown below, where the line through the reference is shown in blue, and the lines through (d) overexposed and (e) low-dose are shown in orange.

Example projection images of the simulated overdose and low-dose scans are shown in Fig. 5a, 5b, and 5c. Line plots along the lines indicated in the projection images are shown in Fig. 5d (reference in blue, overexposed in orange) and in Fig. 5e (reference in blue, low-dose in orange). The desired effects of detector saturation at the skin-air border in the overexposure scan and photon starvation in the low-dose scan can clearly be observed. Note that, as in real acquisitions, the simulation also results in different value ranges for the low-dose compared to the overexposure and reference scans. Given their different peak voltages, the simulated polychromatic X-ray spectra have different energy ranges. As low-energy rays are more likely to be absorbed, higher values are reached for the low-dose scan compared to the two scans with high dose.

### B. SUBJECT ACQUISITION

After the numerical analysis of the results, real acquisitions with patients were performed. As described earlier in Section II, the proposed method requires two scans using an AEC and a low-dose protocol. Acquisitions were performed using a C-arm CBCT system (Artis zeego, Siemens AG, Forchheim, Germany) with a large flat-panel detector (30×40 cm) under an IRB-approved protocol. All projection images were obtained with 14 bits of gray-scale resolution. Each of the 248 projection images had a resolution of 1240×960 pixels and a pixel spacing of 0.308 mm after 2×2 binning. Each scan requires an 8-second gantry rotation time plus a turnaround time of 1.5–2 seconds between rotations for the two scans. Thus, the time cost of the additional low-dose scan is approximately 10 seconds.

A female osteoarthritis patient (69 years, 71 kg) was imaged using the AEC and low-dose protocol while lying supine without moving between the scans. A healthy male subject (27 years, 75 kg) was scanned in the same position but with the difference that movement between the AEC and low-dose scans was simulated by instructing the subject to put the legs upright and place them back on the table without positional guidance between the two scans. This process was repeated and a second low-dose scan was acquired. Example projection images of the AEC scan overlaid with the two low-dose scans are shown in Fig. 3. It is noticeable that for one of the low-dose scans, the subject placed his legs in a similar position as for the AEC scan leading to a mild motion between the scans (Fig. 3a). For the other low-dose scan, the new position of the legs was less similar to the AEC scan leading to a more severe motion between the scans (Fig. 3b).

As depicted in Fig. 4, during the AEC scan the tube outputs (i.e., the tube voltage and tube current-time product per projection) were modulated by the AEC system as a function of the projection number [28], [29]. While the gantry of the C-arm CT system rotated around the two knees together, the AEC elevated the tube outputs along the lateral direction at the beginning and end of the scan, and reduced the output in the anterior-posterior direction where the legs do not overlap.

During the low-dose scan, the values of the tube voltage and the tube current-time product were kept constant at 60 kVp and 0.21 mAs/frame, respectively, which were set up to be able to image soft tissue up to 3 cm deep without causing photon starvation.

### C. SUBJECT REFERENCE DATA

**FIGURE 6.**
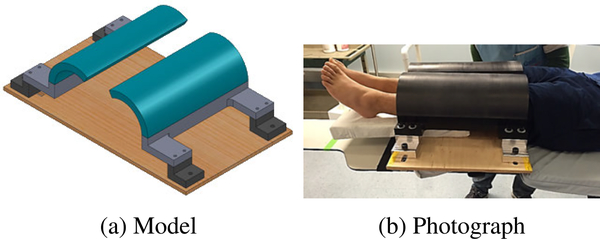
(a) Model and (b) photograph of the beam attenuator used to avoid detector saturation in border regions.

To be able to assess whether the proposed projection combination approach correctly restores the overexposed regions, reference data without overexposure artifacts is required. This can only be realized with the help of external equipment. In our study, we used an apparatus consisting of plastic beam attenuators placed around the knees that prevent saturation by attenuating the X-rays before they reach the patient (see Fig. 6). Thus, reference data without detector saturation artifacts in the knee border region were acquired using an AEC-enabled high-dose acquisition protocol with 496 projection frames.

The two arc-shaped attenuators were designed to provide saturation-free images of the irregular knee shape by attenuating the X-ray beam at the boundary regions. Their positions can be freely adjusted in order to adapt to general populations with various body shapes. The attenuation block is made of Delrin™(acetal homopolymer, density of 1.42 g/cm^3^) and is 25.4 mm-thick. When a beam of 120 kVp passes through the attenuator, only approximately 63% of the photons transmit through it, thereby limiting the maximum number of photons that can reach the object. The transmission rate computation is based on the NIST data [30] for X-ray interaction cross-sections and material properties.

**FIGURE 7.**
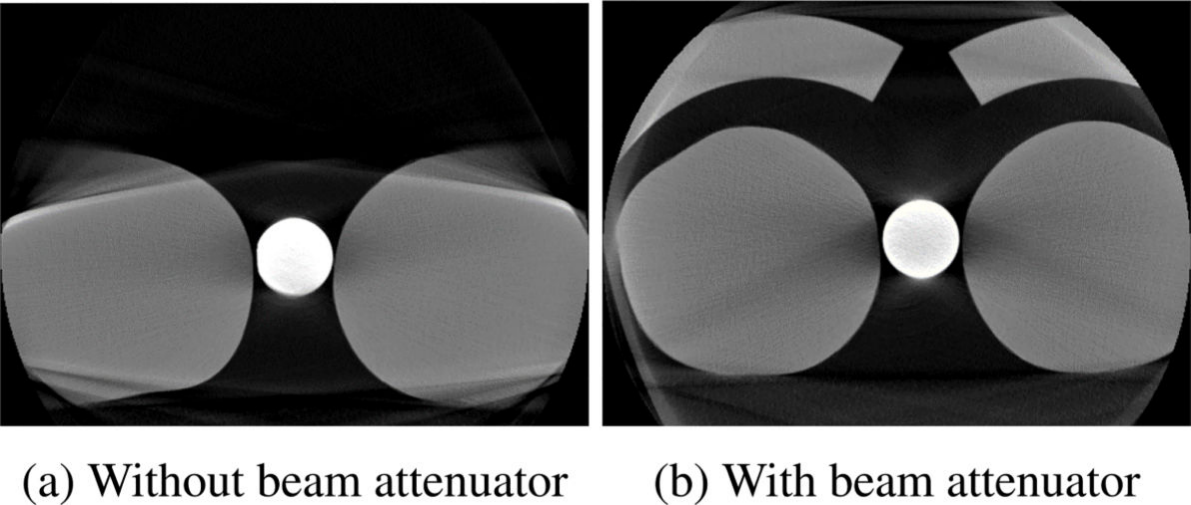
Slices through water bottle volumes reconstructed from 496 AEC-enabled high-dose projections. (a) and (b) show representative central slices of the scans without and with the beam attenuator, respectively. The circular rod phantom simulating cortical bone was placed between the water bottles in order for the built-in AEC system to realistically increase the tube outputs.

In order to verify that the images obtained using the attenuators are suitable as a reference, two 1.5-liter bottles of standard size (diameter: 92 mm) filled with water simulating two legs were scanned. A circular rod phantom of density 1.19 g/cm^3^ was placed between the water bottles to simulate the cortical bone in the leg and to realistically increase the tube outputs operated by the built-in AEC system. Fig. 7a shows a representative central slice of the two water bottles scanned without the beam attenuator, and the resulting artifacts severely degrade the image quality at the border of the bottles. In contrast, when the beam attenuator was implemented, the circle-shaped boundary line of the water bottles was better recovered with uniform HU values throughout the axial surface of the water bottles, as shown in Fig. 7b. Note that although the beam attenuator effectively suppressed saturation artifacts, the use of the beam attenuator could introduce additional artifacts (e.g., beam hardening artifacts and truncation artifacts).

## IV. RESULTS

### A. NUMERICAL SIMULATION

**FIGURE 8.**
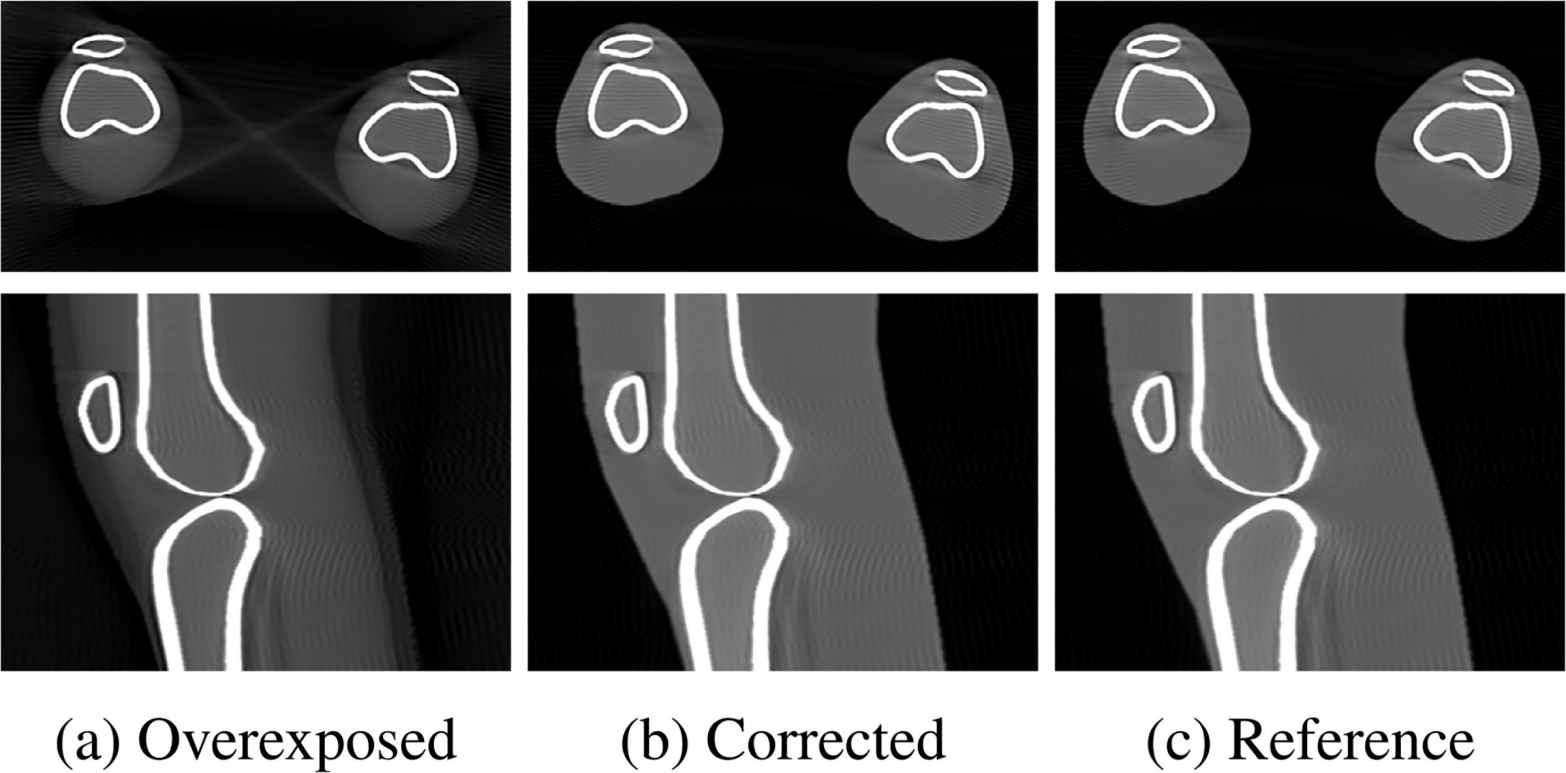
Axial (top) and sagittal (bottom) slices of the reconstructed volumes from (a) overexposed, (b) corrected and (c) reference projection images. Saturation artifacts visible in the overexposed reconstruction are corrected by the proposed approach leading to a similar reconstruction as the reference without overexposure.

Limiting the detector’s dynamic range in the simulation caused several saturation artifacts in the 3D volumetric images when scanning two knees, as shown in Fig. 8a. By applying the proposed projection combination method, the blurred-out skin-air interface of the two legs was recovered and the shape and density of the peripheral regions of the legs became very similar to those in the reference (compare Fig. 8b and 8c).

The qualitative improvement visually observed in the reconstructed images was confirmed by the quantitative analysis in terms of the structural similarity index (SSIM) (0.883→0.999) and root mean squared error normalized by the image value range (nRMSE) (0.055 → 0.003) between the images.

### B. SUBJECT EXPERIMENTS

#### 1) MOTION-FREE CASE

**FIGURE 9.**
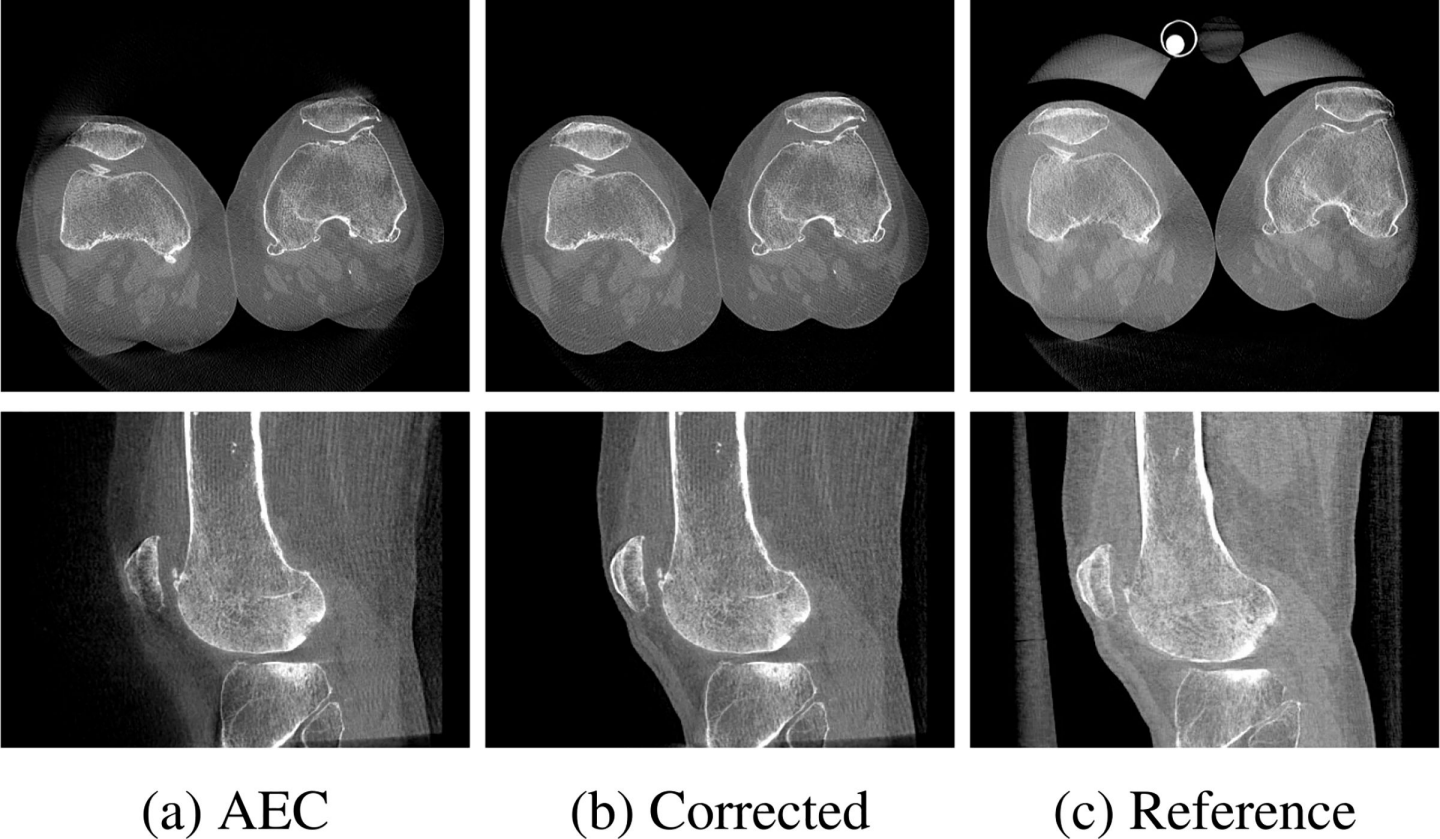
Exemplary axial (top) and saggital (bottom) slices of the reconstructed volumes for the subject without motion between AEC and low-dose scans. (a) Detector saturation artifacts in the AEC scan are mainly visible in the anterior and posterior region of the knee. (b) The proposed projection combination approach restores the saturated regions and produces reconstructions similar to the (c) reference approach with beam attenuators.

In the motion-free case of the first subject, the image projection combination was applied without the need of volume registration. As depicted in Fig. 9a), detector saturation mainly degrades the image quality near the skin-air interface of the knee periphery, resulting in loss of critical tissue structures for clinical diagnosis (e.g., the patella, quadriceps tendon, and patellar tendon) along the boundary of the front and back of the knee.

The proposed projection image combination approach successfully restored these artifacts for the first subject who did not move between the two scans (see Fig. 9b). To evaluate whether the image content is correctly restored, the corrected reconstructed volume is compared to the reference scan acquired using beam attenuators. As a result of the mounting of the attenuators, the subject had to be repositioned for this scan, so it is not possible to show the same slices as for the AEC and corrected scan and only a qualitative comparison is presented. When comparing the corrected slices in Fig. 9b with the reference slices in Fig. 9c, it can be observed that the structures at the skin-air border are properly restored and the patella and patellar tendon have similar morphology and density.

#### 2) MOTION CASE

The processing of the scans of the second subject with intentional motion between the scans included the 3D non-rigid alignment of AEC and low-dose scans.

**FIGURE 10.**
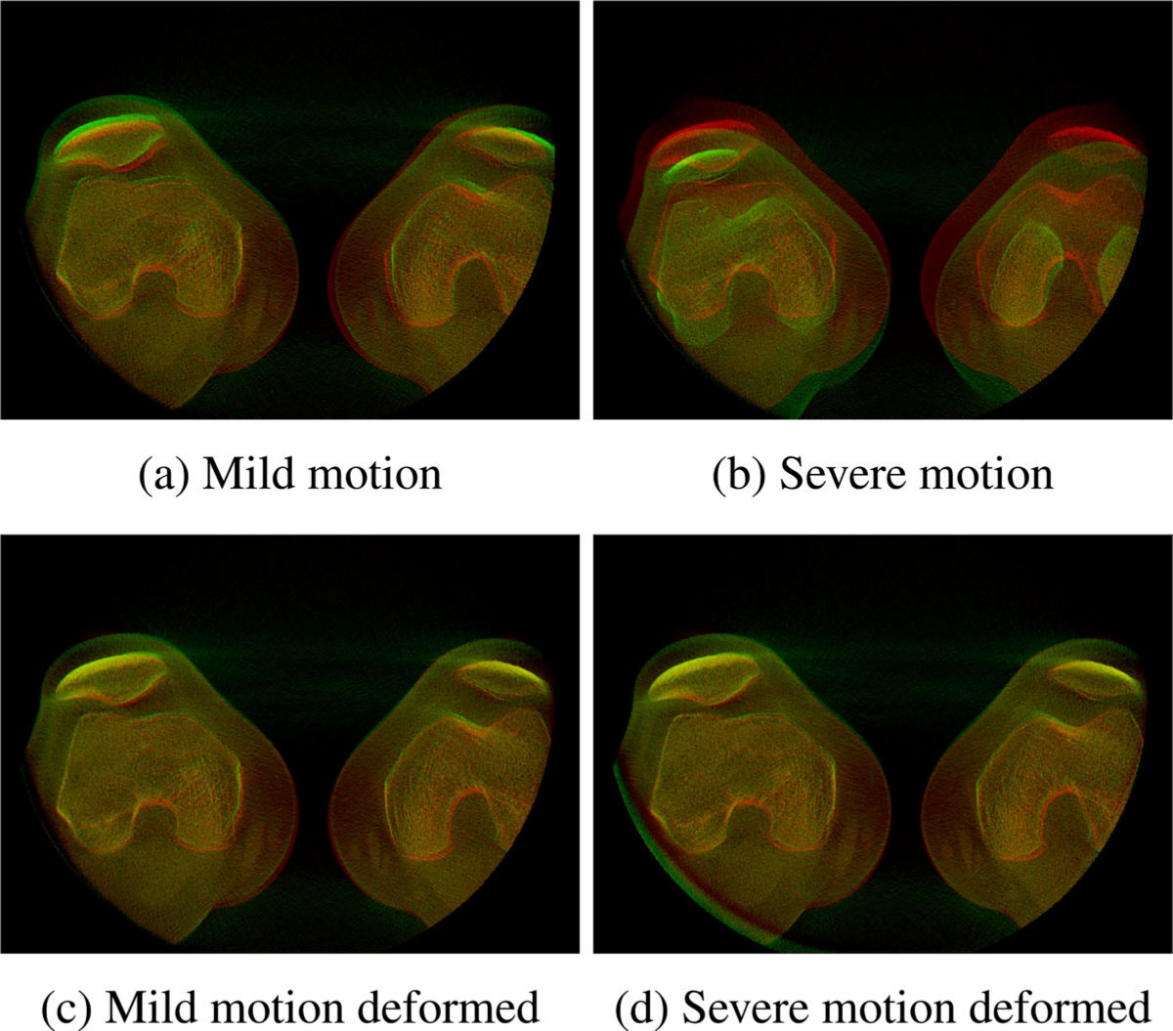
Overlay of example reconstructed slices of the AEC and low-dose scans. In all overlays, AEC images are depicted in red, low-dose images in green, and similar pixels are shown in yellow. The left column shows the mild motion case and the right column the severe motion case. In the top row, the original low-dose reconstructed volumes are overlaid with the AEC volume and the non-rigid motion between the volumes is clearly observable. In the bottom row, the volumes resulting from the deformed reconstruction of the low-dose scan in the first step of the proposed backward-forward-projection process are overlaid with the AEC volume.

**FIGURE 11.**
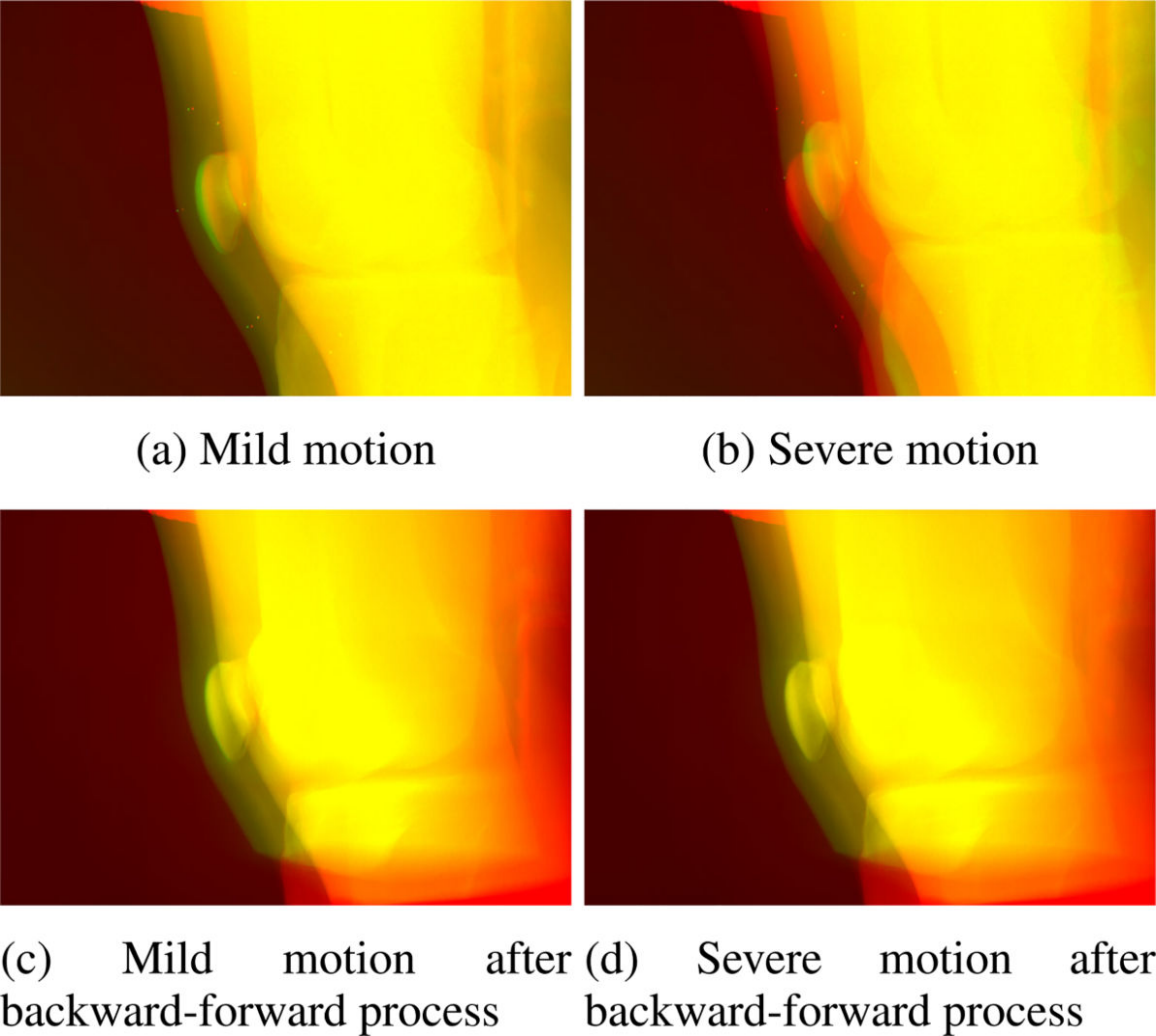
Overlay of example projection images of the AEC and low-dose scans. In all overlays, AEC images are depicted in red, low-dose images in green, and similar pixels are shown in yellow. The left column shows the mild motion case and the right column the severe motion case. In the top row the original low-dose projection images are overlaid with the AEC volume (same as in Fig. 3) and the motion between the projections is visible. In the bottom row, the projection images generated by forward projection in the second step of the proposed backward-forward-projection process are overlaid with the AEC projection images.

The results of the proposed backward-forward-projection process of the low-dose scans creating registered projection images are shown in Figs. 10 and 11. The motion between the AEC scan and the two low-dose scans that was already visible in the projection images (Fig. 3) also leads to non-matching reconstructed volumes if no registration or deformation is applied. This can be observed in the overlay images of a slice through the AEC reconstructed volume (red) with slices through the reconstructed volume of the mild and severe motion low-dose scans (green) in Figs. 10a and 10b. The first step of our proposed approach, namely the deformed reconstruction of the low-dose projections based on tracked marker positions, leads to reconstructed volumes that are well-registered to the AEC volume in the mild as well as severe motion case (see Figs. 10c and 10d). Through the forward-projection in the second step of the proposed approach, projection images of the low-dose scan are generated that better fit with the AEC projection images (Figs. 11c and 11d) than the original low-dose projection images (Figs. 11a and 11b).

**FIGURE 12.**
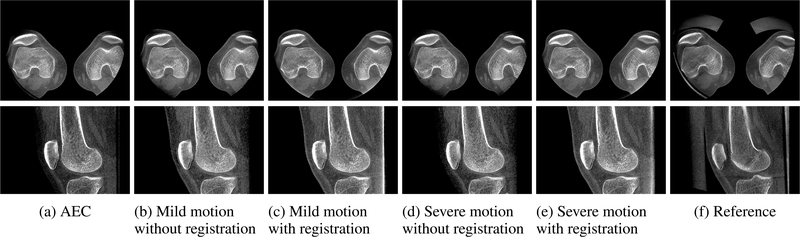
Exemplary axial (top) and saggital (bottom) slices of the reconstructed volumes for the subject with motion between AEC and low-dose scans. (a) Detector saturation artifacts are observable for the AEC scan in the anterior region of the knee. If no registration is applied, the projection image combination fails for (b) mild and (d) severe motion between the AEC and low-dose scans. In both cases of (c) mild and (e) severe motion, the proposed backward-forward-projection approach enabled the effective application of the projection image combination approach and the saturated regions are restored. Similar reconstructions like the (f) reference approach with beam attenuators can be produced using the proposed methods.

The projection images generated in the backward-forward-projection process are then used for the proposed projection combination approach. The results are shown in Fig. 12. Axial and sagittal slices through the volume reconstructed from the AEC scan show similar saturation artifacts as those for the motion-free subject. Once again the structures in the anterior region of the knee joint like the patella and patellar tendon are not entirely visible (Fig. 12a). If no registration is applied before combining the AEC and low-dose scan projection images, these artifacts could not successfully be corrected (see Figs. 12b and 12d). If the registered projection images are used for the projection image combination approach, the missing information can be properly restored, regardless of whether the low-dose scan with mild motion (Fig. 12c) or with severe motion (Fig. 12e) is processed. As for the first subject, only a qualitative comparison with similar slices of the reference scan acquired with beam attenuators is feasible (Fig. 12f). The image regions restored by the proposed approach show a high similarity in shape and values to those of the reference reconstruction.

### C. DOSE ESTIMATION

The effective radiation doses of the over-dose and low-dose scans based on the ICRP 103 tissue-weighting coefficient were calculated as 0.1481 mSv and 0.0067 mSv, respectively. This means that the increment of dose associated with the additional low-dose scan was merely 4.5% of the original dose. In cases without motion between the scans, the dose could be even further reduced by acquiring only a partial-view scan in the lateral views. The associated dose increase would then reduce to 0.0042 mSv corresponding to 2.8%.

## V. DISCUSSION AND CONCLUSION

The proposed saturation correction method effectively suppressed detector saturation artifacts and is even applicable in the case of motion between the two scans.

The simulation successfully reproduced the complex phenomenon of photon starvation in dense regions of the knee as well as the non-linearity in dose-response (i.e., saturation) at the skin-air interface of the knee. However, some limitations of the simulated model need to be discussed. The simplified knee joint model consists of representative tissues of the lower body including four articulating bones (the femur, tibia, patella, and fibula) with bone marrow and fat tissue. Tendons, ligaments, muscle, and other soft tissues of the knee joint are missing. Therefore, the effect of saturation artifacts on the missing tissues is not accounted for. However, compared to bones, soft tissues have a much smaller effective atomic number and lower physical density. Consequently, the measured values among the soft tissues are not noticeably different. The density of fat, muscle, and bone is 0.91, 1.00, and 1.65–1.85kg/m^3^ at 100 KeV, respectively [31]. Therefore, the saturation artifacts that are likely to occur in other tissues can be, to some extent, inferred from fat tissue. On the simulated projections, our proposed projection combination approach reduced saturation artifacts considerably and restored image quality to an extent that was comparable to the reference scan, which is reflected in the high SSIM and low nRMSE scores.

The transfer from simulation to real data was successful. The visual comparison of the results suggests that our method accurately restores the overexposed regions of the knees. However, no quantitative comparison can be presented. This means that it is not guaranteed that the material densities restored by our proposed approach are the same as in the reference scan acquired using beam attenuators. It might be possible to apply a non-rigid registration as in our proposed non-rigid alignment. However, this was not possible in the presented analysis because no markers were placed on the first subject. In the second case, markers were attached to the subject, but the attenuators touched and dented the subject’s legs, leading to deformations too complex to be modeled by our proposed deformed reconstruction based on only few markers placed on the front of the knee.

One drawback of our proposed method might be the manual input parameters such as the bin width and bin distance threshold for the background peak detection or the limit value for removing speckle from the generated masks. The values for these were chosen to work with the simulated and real datasets evaluated in this small study. They could be also be determined via a data-based study with a larger number of subjects.

For the low-dose image acquisitions, the AEC system was deactivated and the values of the tube current-time product and the tube voltage were fixed at 0.21 mAs/frame and 60 kVp, respectively. These values were heuristically chosen in this study and are considered very low compared to those for a standard AEC-enabled scan. To determine these settings, first, the depth of the overexposed soft tissue in the AEC-enabled scan was determined to be approximately 3 cm. Then, a dose just high enough to produce photons that can pass through this tissue depth without causing photon starvation was chosen. The incremental dose associated with the additional low-dose scan of 0.0067 mSv (4.5% of the original dose) is comparable to one-third that from a chest X-ray acquisition or to 0.2% of the annual radiation exposure from natural sources of a resident of the United States (3.2 mSv) [32]. In other words, the incremental dose from the additional low-dose acquisition can be considered negligible.

Even though the additional time and dose required for the low-dose scan are within reasonable limits, it is still cumbersome to scan twice. Dual-energy CT techniques like fast kV-switching [33], [34], photon counting detectors (PCD) with dual-energy capabilities [35], [36] and spectral CT based on PCD [37] may be compatible with the proposed method if the low-dose acquisition can be controlled frame by frame. Admittedly, such advanced technology will not be feasible for widespread clinical use in the near future.

The beam attenuators used to generate reference data attenuate photons along the peripheral regions of the lower extremities such that the number of photons reaching the detector is bounded within the dynamic range of the detector. In previous studies, modeling clay was wrapped around both knees together to prevent artifacts in the same way as the attenuators [3], [38]. However, the use of modeling clay was not ideal for this purpose, as putting weight on the knees could make patients uncomfortable, especially patients with knee pain. Furthermore, knee kinematics and patellofemoral alignment might be altered by the clay, which could lead to the inaccurate diagnosis of knee conditions. These drawbacks are overcome using the attenuators. However, compared to our proposed methods, the attenuators have potential limitations. The size of the attenuators is limited by the C-arm rotation trajectory. Some of the taller or heavier subjects complained of discomfort during the process of fitting their legs within the attenuators. Furthermore, thick attenuators with sharp edges could introduce beam hardening and aliasing artifacts, and truncation artifacts could occur because the beam attenuators are cut off in the field of view at most gantry angles. Note that we acquired 496 views for the reference scans to achieve a high-quality image. If the number of projections is reduced to 248 as for the scans without attenuators, view-aliasing artifacts may occur, leading to poor image quality compared to that of our proposed approaches with the same number of views.

Lower body motion between the AEC and low-dose scans hinders the application of the proposed projection combination method. The motion that occurred when the legs were raised up vertically and placed back on the table in this study is assumed to be considerably larger than the magnitude of movement that can occur in reality. Nonetheless, image data with a high amount of motion are clinically meaningful, because knee patients are likely to show more involuntary movements and motion will also be more severe when imaging under weight-bearing conditions.

The proposed non-rigid inter-scan registration algorithm of low-dose to AEC effectively reduced the mis-alignment of the projection images, making them usable for projection image combination. However, it has to be noted that the backward-forward-projection approach introduces some inaccuracies in the data. The filtered back-projection adapted in this work can only give approximate results for CBCT, which leads to an information loss of the generated projections compared to the original projections [23]. Furthermore, factors like truncation, finite detector and reconstruction resolution, and the coverage of the scanning volume will have an influence on the generated projection images. In the generated projection images in Figs. 11c and 11d, there are missing parts at the top and bottom of the images that occur owing to the conebeam coverage of the CBCT scan. In these regions, only the original projection image (shown in red) is visible. Many of these inaccuracies, however, only have an influence on the border regions of the projection images. As the knee joint as region of interest is placed to be displayed in the center areas, the information is sufficiently preserved throughout the backward-forward-projection.

The accuracy of the deformation during back-projection depends on the placement and number of the metallic markers. In the current configuration, the markers were only placed on the front of the knee joint. Therefore, the resulting reconstructed volumes in Figs. 10c and 10d match the AEC volumes in the anterior region and at the patella well. However, larger deviations can be observed at the femoral bone and posterior parts of the knees. As the goal of the current analysis was to restore information mainly in the anterior region, the registration was sufficiently accurate. However, in future investigations and also when applying the proposed method to other cases suffering from overexposure, the number of markers and the marker placement should be thoroughly considered.

The presented evaluation on numerical simulations and a small set of scans yields promising results. Based on these findings, the acquisition and processing parameters for the proposed approach can be optimized in a larger study.
